# Supplementary material for: Towards Sustainable and Nutritionally Enhanced Flatbreads from Sprouted Sorghum, Tapioca, and Cowpea Climate-Resilient Crops
Source: Foods. 2023 Apr 13;12(8):1638. doi: 10.3390/foods12081638 (PMC10138180; doi:10.3390/foods12081638)
Supplement: Supplementary file 1 [file foods-12-01638-s001.zip › foods-2301745-supplementary.pdf]

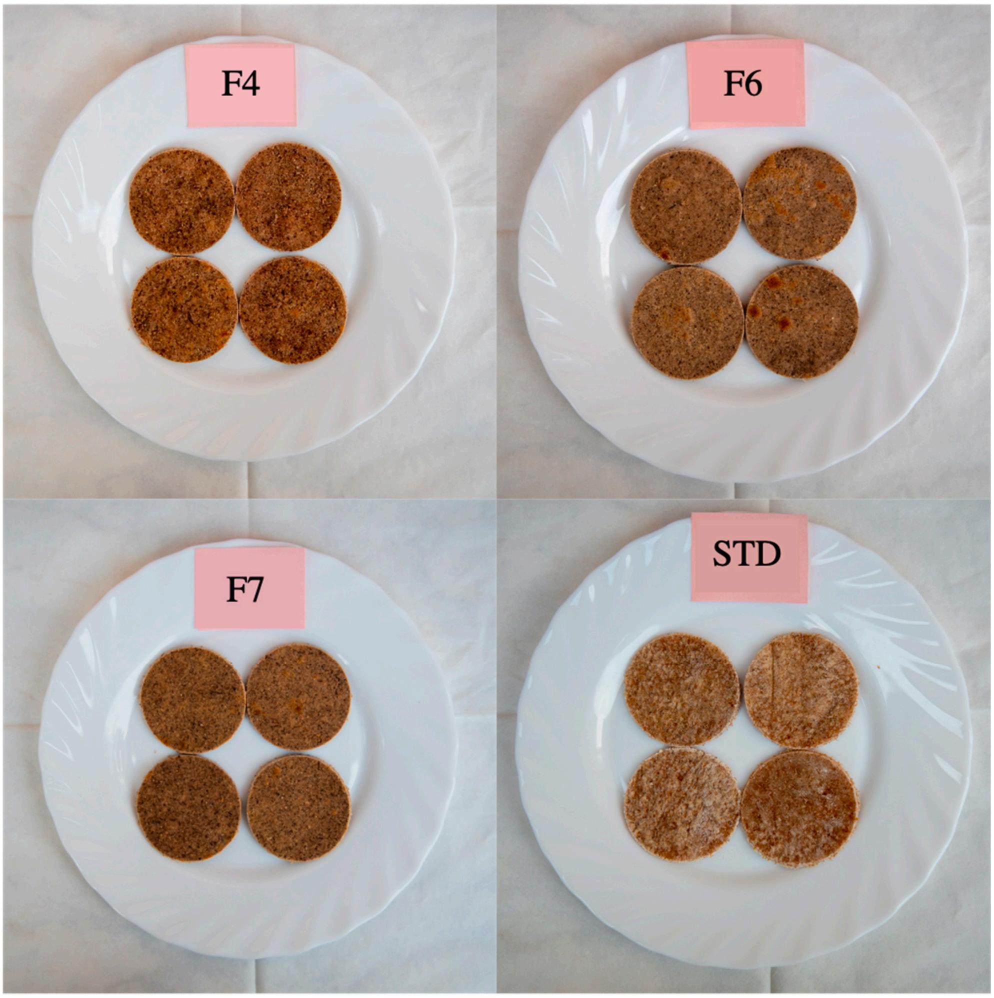

Figure S1. Selected composite flour flatbreads based on technological (F6), nutritional (F6 and F7) and economic sustainability criteria (F4). STD, standard 100% W bread.

Table S1: R^2 and Q^2 of the models generated by the controlled responses.

Responses	R^2	Q^2
P_f	0.9	0.2
P_e	0.7	0.5
E_f	0.7	0.2
E_e	0.6	0.5

P_f, force at rupture from puncture test; P_e, extensibility from puncture test; E_f, force at rupture from one-dimensional extensibility test; E_e, extensibility from one-dimensional extensibility test.

3.1.1. Significance of the models generated for the controlled responses

The model generated for the P_f response was characterized by an $R^2 = 0.9$ and a $Q^2 = 0.2$. A probable linear dependence of the response on the SS and C factors was observed: an increase in SS materialized in a probable negative effect on the response (decrease in P_f), while an increase in C was more likely to affect positively (increase in P_f). A negative effect from the interaction of the two factors (SS * C) was also possible.

The model generated for the P_e response was characterized by an $R^2 = 0.7$ and a $Q^2 = 0.5$. A probable linear dependence on factors T and W was observed: an increase in T materializes in a probable positive effect on the response (increase in P_e), while an increase in W more likely affected negatively (decrease in P_e). The interaction coefficients revealed a positive quadratic dependence of the response on the factors W and T (W * W and T * T) indicating the need for a second order equation to describe the model. There was also an interaction effect of the two factors (W * T), which was manifested by a distortion effect of the response surface.

The model generated for the E_f response was characterized by an $R^2 = 0.7$ and a $Q^2 = 0.2$. A probable linear dependence on the factors SS, T and water was found: an increase in SS or T materialized in a probable negative effect on the response (decrease in E_f), while the increase in water was more likely to affect positively (increase of E_f). As described before, also in this case the interaction coefficients revealed a positive quadratic dependence of the response on the factors SS and T (SS * SS and T * T).

Finally, the model generated for the E_e response was characterized by an $R^2 = 0.6$ and a $Q^2 = 0.5$. Only the factor SS exerted an influence on the response, and a negative linear dependence was identified for it (as SS increased, E_e decreased).

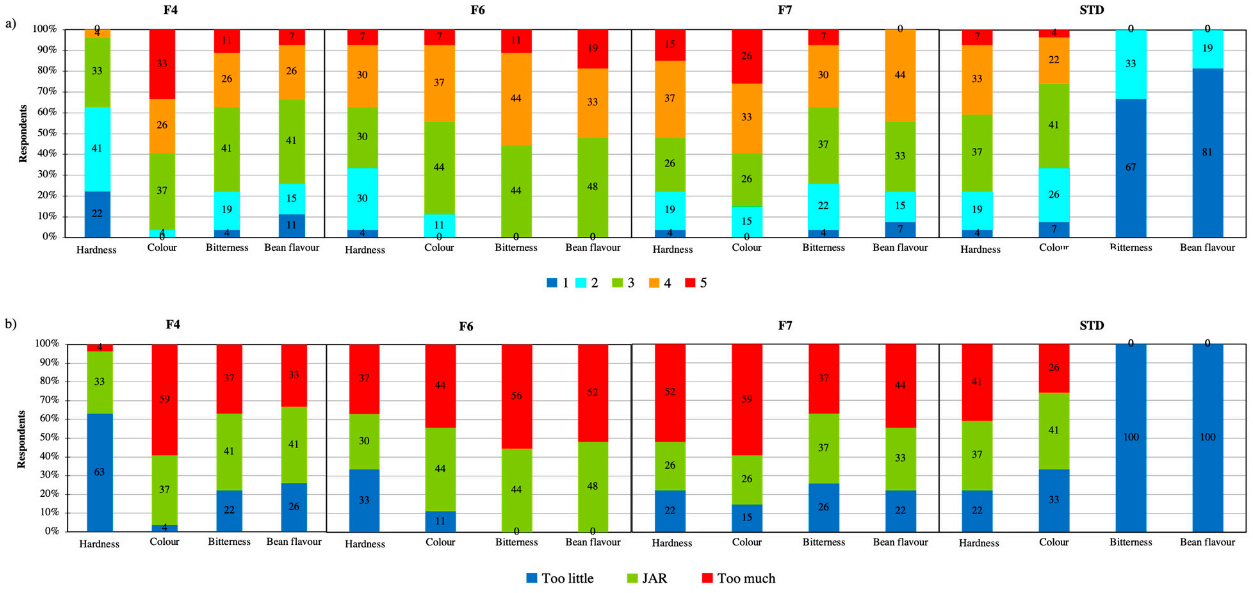

Figure S2. Percentage of respondents (n=27) giving consumer ratings for breads' hardness, darkness, bitterness and bean flavour a) based on 5-point JAR scale; b) collapsed on a 3-level scale.

Table S2. Penalty analysis table for F4 bread[a].

Variable	Level	Frequencies (%)	Sum (Overall liking)	Mean (Overall liking)	Mean drops	Penalties	*p*-value
Hardness	Not enough	62.96	96.00	5.65	0.13	0.11	0.790
	JAR	33.33	52.00	5.78			
	Too much	3.70	6.00	6.00	-0.22		
Darkness	Not enough	3.70	6.00	6.00	-0.40	-0.17	0.686
	JAR	37.04	56.00	5.60			
	Too much	59.26	92.00	5.75	-0.15		
Bitterness	Not enough	22.22	33.00	5.50	0.50	0.50	0.204
	JAR	40.74	66.00	6.00			
	Too much	37.04	55.00	5.50	0.50		
Bean flavour	Not enough	25.93	41.00	5.86	0.05	0.34	0.383
	JAR	40.74	65.00	5.91			
	Too much	33.33	48.00	5.33	0.58		

Frequency of responses for the 3-level scale; sum of the liking scores for each level; average liking for each level. Mean drop for the "too much" and "too little" levels shows how many points of liking were lost for having a product that was "too much" or "too little" compared to the JAR level; penalty shows how many points of liking were lost for not being

as expected by the consumer. The *p*-value (significance level = 0.05) tested whether the penalty was significantly different from 0.

Table S3. Multiple pairwise comparisons (Nemenyi's procedure) performed on second preference ranking test data.

Sample	Rank Sum of Sample*	Rank Mean of Sample
F7	39c	1.44
F6	63bc	2.33
F4	67b	2.48
STD	101a	3.74

* Rank sum of the sample = $\sum$(number of panelists $\times$ respective rank position). Lower rank sum indicated better-liked samples.
